# Serotype-specific and temperature-dependent biofilm formation in *Salmonella*: Limited impact of antimicrobial resistance or source

**DOI:** 10.1016/j.bioflm.2026.100360

**Published:** 2026-03-17

**Authors:** Jun Lv, Tingting Ju, Wenlin Ye, Changlin Yu, Qi Cheng, Haitang Xiong, Xinyun Fan, Lanfang Liu, Zhiyong Song, Bin Luo, Yonghong Zhang

**Affiliations:** a Department of Cardiology, Hubei Provincial Clinical Research Center for Precision Diagnosis and Treatment of Liver Cancer, Hubei Key Laboratory of Embryonic Stem Cell Research, Taihe Hospital, School of Basic Medicine, Hubei University of Medicine, Shiyan, 442000, China; b Shiyan Key Laboratory of Medicinal Plants and Evolutionary Genetics, Hubei Key Laboratory of Wudang Local Chinese Medicine Research, Hubei University of Medicine, Shiyan, 442000, China; c Health Science Center, Yangtze University, Jingzhou, China; d Shiyan Center for Disease Control and Prevention, Shiyan, 442000, China; e State Key Laboratory of Agricultural Microbiology, College of Science, Huazhong Agricultural University, Wuhan, 430070, China

**Keywords:** *Salmonella*, Biofilm, Asymptomatic carriers, Serotype, Drug resistance

## Abstract

**Objective:**

*Salmonella* is a major zoonotic and foodborne pathogen whose ability to form biofilms enhances antimicrobial tolerance and environmental persistence. This study aimed to investigate how temperature-dependent biofilm formation is influenced by serotype, multidrug resistance (MDR) status, and source of isolation.

**Methods:**

A total of 114 *Salmonella* isolates from food and humans (including clinical cases and asymptomatic carriers) were assessed for biofilm-forming capacity. Biofilms were stained with crystal violet and quantified by measuring the OD_570_ after incubation at 15 °C, 25 °C, and 37 °C for 3 days. Isolates were categorized into weak, moderate, and strong biofilm formers. Associations with serotype, MDR status, and isolation source were analyzed using ANOVA and multiple regression. PCR screening was performed to examine the distribution of 28 biofilm-related genes.

**Results:**

Biofilm formation peaked at 25 °C across all sources, indicating a strong temperature effect. Distinct serotype-specific patterns of biofilm capacity were observed, while antimicrobial resistance and source showed no significant associations. Statistical analysis identified temperature (F = 36.46, *p* = 6.40 × 10^−15^) and serotype (F = 3.31, *p* = 6.91 × 10^−8^) as primary determinants of biofilm variability. Among the 28 genes screened, 24 were universally present, whereas the distribution of *rcK*, *fimH*, *steB*, and *pefA* was serotype-dependent but independent of extent of biofilm formation.

**Conclusions:**

Biofilm formation in *Salmonella* was primarily driven by temperature and serotype, while no significant association was observed with antimicrobial resistance or isolation source in this study. These findings provide new insights into the ecological adaptation of *Salmonella* biofilms and have implications for controlling persistence and transmission under fluctuating environmental conditions.

## Highlights

• Biofilm formation in *Salmonella* is enhanced at 25 °C across all sources.
• Serotype and temperature mainly determine biofilm-forming capacity.
• Antimicrobial resistance and source show little effect impact on biofilm formation.
• Most biofilm genes (23/28) are universally present in all *Salmonella* isolates.
• Genes like *rck* and *fimH* are serotype-linked, not related to biofilm strength.

## 1 Introduction

Salmonellosis is a serious foodborne disease caused by *Salmonella* infection. Approximately 200 million people worldwide suffer from salmonellosis each year, with over 200000 deaths from the disease [1]. In recent years, although the incidence rate of salmonellosis has declined with the improvement of sanitary conditions and food safety, it still poses a health threat to the public, especially in developing countries (GBD 2017 Typhoid and Paratyphoid Collaborators). The World Health Organization (WHO) ranks *Salmonella* among the four leading causes of human diarrheal illness worldwide [2]. More than 2600 *Salmonella* serotypes have been reported according to the White-Kauffmann-Le Minor scheme, but less than 100 serotypes are known to cause infections in humans [3,4]. *Salmonella* enterica serovars are primarily classified into two distinct pathotypes. Typhoidal serovars (*S. Typhi* and *S. Paratyphi A, B, C*) cause the systemic infection of enteric fever, whereas non-typhoidal serovars (NTS) typically cause self-limiting gastroenteritis.

*Salmonella* not only causes acute infections, but can also lead to chronic asymptomatic carriage in a subset of patients [5]. The acute manifestations of *Salmonella* infections primarily include gastroenteritis and enteric fever. An asymptomatic or chronic carrier is defined as an individual who harbors a persistent *Salmonella* infection, typically in the gallbladder, biliary tract, or intestinal epithelium, without exhibiting clinical symptoms [6]. This state can last for months to years, during which the carriers may intermittently shed the bacteria in their stool, serving as a reservoir for transmission to others [7,8]. Cases of chronic carriers of typhoidal salmonellosis (caused by *Salmonella* Typhi and Paratyphi) are frequently reported and well-documented, particularly in endemic regions. Approximately 3-5% of individuals infected with *Salmonella* Typhi develop into chronic carriers, with the gallbladder serving as the primary reservoir for bacterial persistence[9,10]. In contrast, chronic carriage of nontyphoidal salmonellosis (NTS) is relatively scarce and poorly understood [6]. The true prevalence of NTS chronic carriers may be underestimated due to limited surveillance, asymptomatic nature, and the lack of routine screening in most healthcare settings. Healthy carriers of *Salmonella* serve as natural reservoirs for the bacteria, playing a critical role in its persistence and transmission within communities. Studies have shown that food workers, such as those in restaurants, catering services, and food processing facilities, exhibit higher carriage rates compared to non-food workers [11]. Asymptomatic *Salmonella* carriers among food handlers pose significant food safety and public health challenges, as they can unknowingly contaminate food products during preparation or handling [12].

Biofilms are structured three-dimensional multicellular communities of microorganisms that attach to natural or synthetic surfaces and grow embedded within a self-produced extracellular polymeric substance (EPS) matrix [13]. This matrix, composed of polysaccharides, proteins, extracellular DNA, and lipids, provides protection to the embedded microbes and under stressful conditions, such as exposure to antibiotics, disinfectants, or host immune responses [14,15]. *Salmonella* biofilms can form on a variety of surfaces, including medical devices, food processing equipment, and biological tissues [16]. The formation of biofilms is a key factor in the persistence and transmission of *Salmonella* [17]. In the food industry, biofilms on equipment surfaces can serve as reservoirs for contamination, leading to foodborne outbreaks. Within the human body, biofilms contribute to chronic infections, particularly in individuals with gallstones or compromised immune systems. Chronic *S. Typhi* infections are primarily localized to the gallbladder, where bacteria form biofilms on cholesterol gallstones [18]. These biofilms enhance bacterial resistance to bile, host immunity, and antibiotics [[19], [20], [21]], and facilitate transmission through the periodic release of bacteria into bile and feces [22]. Biofilm formation is a multifactorial process influenced not only by temperature but also by strain-specific traits, environmental conditions (nutrient availability, osmotic stress, pH, oxygen), surface characteristics, and cell-to-cell signaling mechanisms [23].

However, research exploring the potential relationship between asymptomatic carriage of NTS and its biofilm-forming ability remains very limited, leaving a gap in our understanding of chronic carriage. In this study, we hypothesized that biofilm formation in *Salmonella* is influenced by temperature, serotype, antimicrobial resistance, and source of isolation, with stronger biofilm formation expected at temperatures relevant to the environment and host. To test this hypothesis, we selected three representative conditions: 15 °C (typical outdoor temperature), 25 °C (standard room temperature), and 37 °C (human and mammalian body temperature). We qualitatively and quantitatively evaluated the biofilm formation ability of *Salmonella* strains isolated from food, patients, and asymptomatic carriers in the Shiyan, China using microtiter plate crystal violet staining. By analyzing the relationship between *Salmonella* biofilm formation ability and factors such as serotypes, multidrug resistance, and sources, we aimed to better understand how biofilms contribute to chronic carriage and to provide insights for developing more effective public health strategies.

## 2 Material and methods

### 2.1 Salmonella strains

This study tested a total of 114 archived *Salmonella* isolates. The isolates originated from three sources: food (n = 26), including pork (n = 10), poultry (n = 8), beef (n = 5), salad vegetables (n = 2), and a sandwich (n = 1); asymptomatic carriers (n = 41); and clinical patients (n = 47). Asymptomatic carriers were defined as individuals with no recent symptoms of salmonellosis (confirmed by questionnaire) but with at least one culture-positive stool sample for *Salmonella*. In our previous study, all strains were classified into 31 serotypes, with serovars Typhimurium, Derby, Enteritidis, Thompson, London, Agona, and Gold Coast being the predominant ones [24]. Among them, 53.5% isolates showed resistance to three or more classes of antibiotics, and were defined as multidrug-resistant (MDR) strains. Archived *Salmonella* strains were mixed in Tryptic soy broth (TSB) medium containing 20% glycerol and stored at −80 °C. Before experiment, strains were revived in TSB at 37 °C for 18-24 h.

### 2.2 Biofilm formation and quantitative analysis

Biofilm forming ability of *Salmonella* isolates were tested by 96-well microtiter plate crystal violet staining assay according to previous report [25]. After activation, a single colony from each *Salmonella* isolate was inoculated into 10 mL of TSB and incubated at 37 °C to an OD_600_ of 1.0 (approximately 5 × 10^8^ CFU/mL). For the biofilm assay, 5 μL of this suspension was added to 200 μL of sterile TSB in each well of a 96-well plate, yielding a final inoculum of approximately 1 × 10^7^ CFU/mL to initiate biofilm formation. Each strain was tested in triplicate by dispensing the same inoculum into three independent wells. *Staphylococcus aureus* ATCC 25923, a well-established strong biofilm-forming strain, was used as a positive control to validate the assay, while wells containing only TSB supplemented with 5 μL of PBS served as the negative control. Preliminary experiments were conducted to evaluate biofilm formation at 15 °C, 25 °C, and 37 °C over 1–7 days. Most isolates reached peak formation after 3 days of incubation at 25 °C and 37 °C, whereas peak formation at 15 °C occurred after 5 days. Consequently, a 3-day incubation period was adopted for all subsequent assays to maintain consistent and comparable experimental conditions. The microtiter plates were placed in vented plastic boxes to prevent evaporation and incubated at 15 °C, 25 °C, and 37 °C for 3 days. After incubation, the bacterial suspension was carefully aspirated with a pipette to avoid disturbing the biofilm, ensuring no liquid residue remained. The wells were gently washed three times with 250 μL PBS, and the plate was then air-dried at room temperature to remove residual liquid. After drying, the biofilm in each well was fixed with methanol and stained with 250 μL of 0.1% crystal violet for 30 min. Following removal of the stain and washing with PBS, the bound dye was dissolved in 33% glacial acetic acid. One hundred microliters of solution were transferred to a new well, and optical density (OD) was measured at 570 nm by a microplate reader (Molecular Devices, USA). The threshold optical density (ODc) was defined as the mean absorbance value of OD_570_ plus three standard deviations (SD) of the negative control wells. *Salmonella* isolates were classified based on biofilm formation as follows [25]: non-producer (OD_570_ ≤ ODc), weak producer (ODc < OD_570_ ≤ 2 ODc), moderate producer (2 ODc < OD_570_ ≤ 4 ODc), and strong producer (4 ODc < OD_570_). All experiments were repeated three times.

### 2.3 Screening for genes involved in biofilm formation

Genomic DNA was extracted from *Salmonella* isolates using a commercial DNA extraction kit (Tiangen, Beijing, China) following the manufacturer's protocol. The primer sequences for biofilm-related genes were listed in Supplementary Table S1, which were synthesized by Sangon Biotech (Shanghai). The PCR amplification was performed in a 25 μL reaction volume containing: 12.5 μL of 2 × Es Taq MasterMix (Tiangen), 1 μL of each primer (0.4 μM), 1 μL of genomic DNA template, and 9.5 μL of ddH_2_O. Cycling parameters: 94 °C for 5 min; 35 cycles of 94 °C (1 min), gene-specific annealing temperature (Supplementary Table S1, 1 min), 72 °C (1 min); final extension 72 °C for 10 min. Amplification products were resolved on 1% agarose gels impregnated with GoldView I fluorescent dye and visualized under UV illumination.

### 2.4 Statistical analysis

To assess factors influencing biofilm-forming abilities at different temperatures, quantitative OD_570_ values were analyzed in relation to serotype, antimicrobial resistance profile, and isolation source. Biofilm-forming ability was treated as the dependent variable, while serotype (31 categories), multidrug resistance (MDR vs. non-MDR), and isolation source (food, clinical, asymptomatic carrier) were included as independent variables.

Hierarchical clustering analysis was performed for exploratory visualization of biofilm formation patterns. Log-transformed OD_570_ values [log_1_(OD_570_ + 1)] were used to construct a clustered heatmap. Temperatures were hierarchically clustered based on similarity in biofilm-forming abilities, while strains were ordered by isolation source without row clustering. Heatmaps and clustering were generated in Python using the seaborn library.

Principal component analysis (PCA) was conducted to explore patterns of biofilm formation across different temperatures and to assess clustering by serotype, isolation source, and antimicrobial resistance status. The analysis and visualization were implemented in Python using the scikit-learn, pandas, numpy, seaborn, and matplotlib libraries.

Nonparametric comparisons between groups were performed using the Mann-Whitney *U* test (for two independent samples) with Bonferroni correction for multiple comparisons. All analyses were conducted in Python (version 3.9) with the SciPy (version 1.7.3) and statsmodels (version 0.13.2) libraries. Significance was defined as a corrected *p*-value <0.05. Effect sizes were reported as rank-biserial correlation coefficients (r) for nonparametric tests.

## 3 Results

### 3.1 Overall biofilm-borming ability of 114 Salmonella isolates

**Fig. 1:**
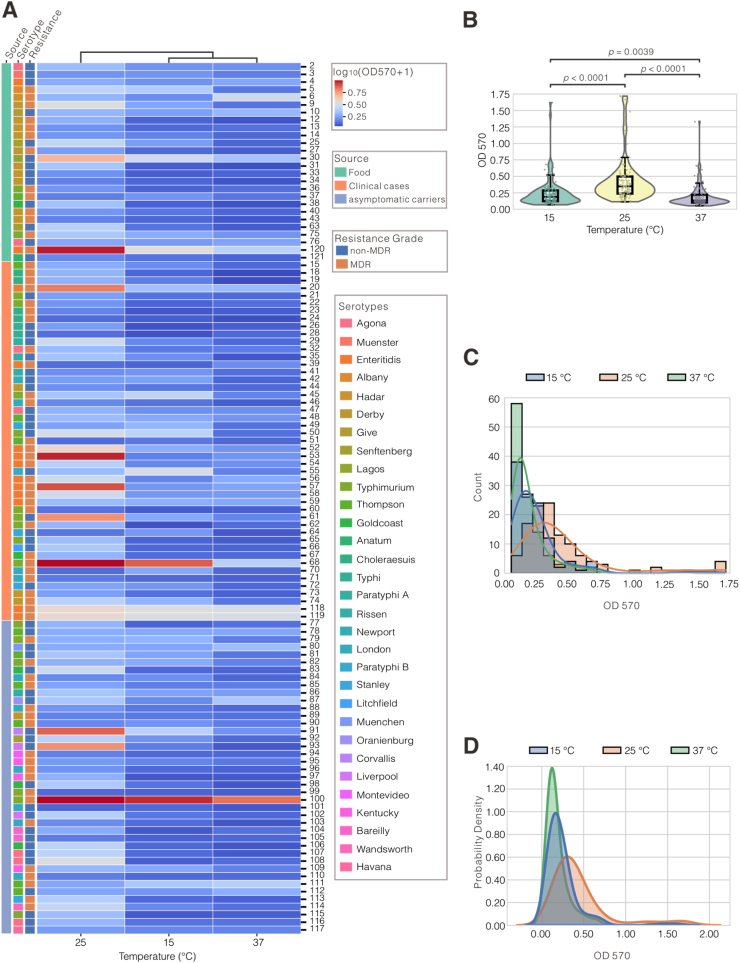
**Temperature-dependent biofilm formation in *Salmonella* strains across source, serotype, and resistance profile. (A)** Heatmap of log10 (OD_570_+1) values representing biofilm formation capacity in 114 *Salmonella* strains grown at 15 °C, 25 °C, and 37 °C. Strains are grouped by source (Food, Clinical cases, Asymptomatic carriers) with adjacent color bars indicating: source (left), serotype (middle), and resistance grade (non-MDR, MDR; right). Temperature conditions are hierarchically clustered (dendrogram). Color intensity reflects relative biofilm formation capacity. Data demonstrate temperature-dependent biofilm modulation across diverse *Salmonella* strains. **(B)** Violin-box plots of OD_570_ values at 15 °C, 25 °C, and 37 °C, showing distribution, overlaid with box plots and individual data points. Statistical comparisons were performed using the Mann–Whitney *U* test with Bonferroni correction for multiple testing. Significant differences were observed as shown. **(C)** Histogram showing the distribution of OD_570_ values at the respective temperatures. Each bar represents the count of isolates. KDE (Kernel Density Estimate) lines are overlaid to visualize the distribution trend for each temperature. **(D)** Single KDE plot shows the distribution of biofilm formation capacity. Shaded areas represent normalized density. Curves illustrate right-skewed distributions and peak biofilm formation at 25 °C. (For interpretation of the references to color in this figure legend, the reader is referred to the Web version of this article.)

The biofilm-forming capacity of 114 *Salmonella* isolates was assessed by measuring OD_570_ values after incubation at 15 °C, 25 °C, and 37 °C (Table 1). The results revealed that biofilm formation showed strong temperature dependence, with significantly higher OD_570_ values observed at 25 °C compared to 15 °C and 37 °C (*p* < 0.0001, Mann–Whitney *U* test, Bonferroni corrected; Fig. 1A and B; Supplementary Tables S2 and S3). Hierarchical clustering further separated 25 °C from the other two conditions (Fig. 1A), indicating a distinct phenotypic response at this intermediate temperature.

**Table 1:** Evaluation of biofilm-forming ability among 114 *Salmonella* isolates at different temperatures.

Serovars	Non-biofilm producers (No.)			Weak biofilm producers (No.)			Moderate biofilm producers (No.)			Strong biofilm producers (No.)		
	15 °C	25 °C	37 °C	15 °C	25 °C	37 °C	15 °C	25 °C	37 °C	15 °C	25 °C	37 °C
Typhimurium				8	1	9	1	3	6	8	13	2
Derby			1	7		12	5	9	1	2	5	
Enteritidis				1			6	1	5	6	12	8
Thompson					1	2	6	2	5	4	7	3
London				5	2	9	3	7		1		
Agona				2		3	5		3		7	1
Goldcoast			1	4		4	1				5	
Choleraesuis			4	3	1		1	1			2	
Kentucky				1		1	1	1	2	1	2	
Senftenberg						3	2			1	3	
Stanley				1		3	2	1			2	
Bareilly				2		2		1			1	
Liverpool						2	2				2	
Paratyphi A						2	2				2	
Paratyphi B									2	2	2	
Rissen						1	1	1	1	1	1	
Typhi				2		2		2				
Albany									1	1	1	
Anatum						1	1	1				
Corvallis									1	1	1	
Give							1				1	1
Hadar							1				1	1
Havana							1	1	1			
Lagos										1	1	1
Litchfield				1		1					1	
Montevideo				1		1		1				
Muenchen										1	1	1
Muenster							1	1	1			
Newport						1				1	1	
Oranienburg							1				1	1
Wandsworth						1	1				1	
Total	0	0	6	38	5	60	45	33	29	31	76	19

To quantify the extent of biofilm formation, we defined a threshold OD_570_ value of 0.346, corresponding to the 75th percentile of all aggregated measurements across strains and temperatures (Fig. 1D). This data-driven threshold distinguishes high biofilm formers from the broader population. At 25 °C, 50.9% of isolates exceeded this threshold, compared to only 14.0% and 10.5% at 15 °C and 37 °C, respectively, highlighting 25 °C as the most permissive temperature for robust biofilm production (Fig. 1D; Supplementary Table S4). Kernel density and histogram analyses (Fig. 1C and D) revealed right-skewed distributions across all temperatures, with 25 °C displaying the broadest distribution and highest density peak (OD_570_ = 1.72). In contrast, biofilm formation was suppressed at 15 °C, likely due to cold stress, and moderately expressed at 37 °C, possibly reflecting host-associated regulatory constraints. Collectively, these findings identify temperature as a key determinant of *Salmonella* biofilm phenotypes, with 25 °C emerging as a critical threshold for enhanced matrix production.

### 3.2 Serotype-dependent and temperature-responsive variation in biofilm formation

**Fig. 2:**
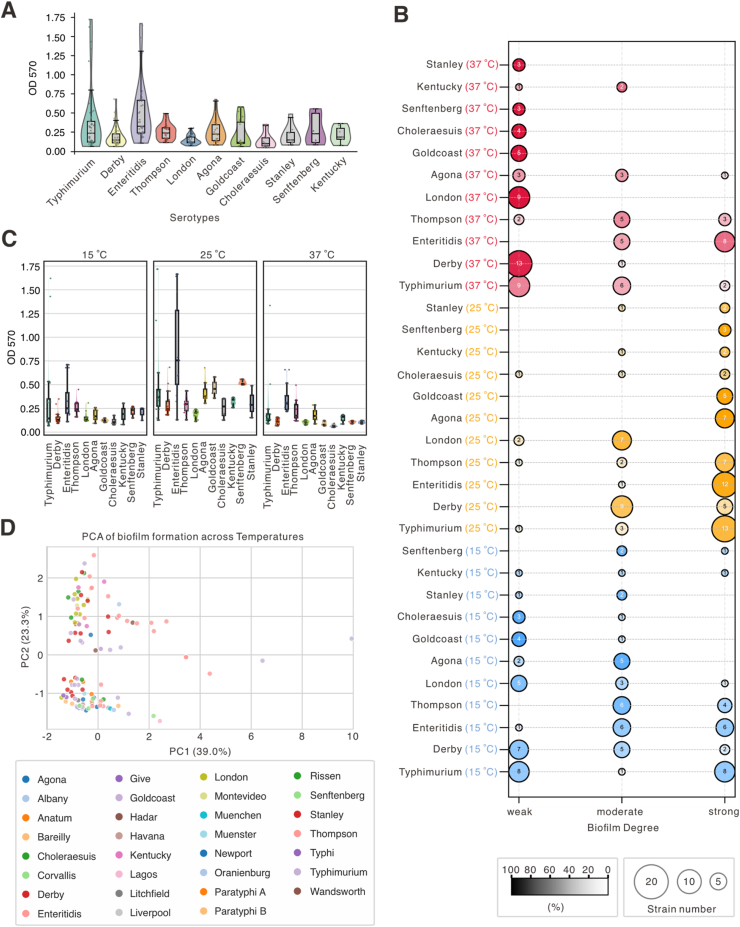
**Association between serotype and temperature-dependent biofilm formation in *Salmonella*. (A)** Violin-box plots showing OD_570_ values for biofilm formation across the top 11 *Salmonella* serotypes (≥3 strains per serotype), ordered by median OD_570_. Boxes indicate interquartile range (IQR), medians, and 1.5 × IQR whiskers; outliers are plotted individually. Statistical comparisons were performed using Mann–Whitney–Wilcoxon tests with Bonferroni correction; significant pairwise differences are detailed in Supplementary Table S4. **(B)** Bubble plots representing the distribution of biofilm-forming categories (weak, moderate, strong) by serotype and temperature (15 °C, 25 °C, 37 °C). Bubble size reflects the number of isolates; y-axis indicates serotype–temperature combinations. Only the top 11 serotypes are shown. **(C)** Violin-box plots of OD_570_ values across the 11 most prevalent serotypes at three temperatures. Each panel overlays violin plots (distribution), boxplots (median and IQR), and individual data points. **(D)** Principal Component Analysis (PCA) of 114 strains based on biofilm formation across temperatures. PC1 (39.0%) distinguishes strains with opposing biofilm formation at 25 °C and 37 °C, while PC2 (23.3%) captures adaptation to low temperature (15 °C), as indicated by the loading pattern.

To evaluate the association between *Salmonella* serotype and biofilm-forming capacity, OD_570_ values were compared across the top 11 serotypes (≥3 strains each). Violin-box plots revealed significant variation in median OD_570_ among serotypes (Fig. 2A; Supplementary Table S5), with *S. Enteritidis* displaying the highest median biofilm formation (0.331), significantly higher than *S. Derby*, *S. London*, *S. Thompson*, and *S. Choleraesuis* (adjusted *p* < 0.05; Supplementary Table S5). In contrast, *S. Choleraesuis* exhibited the lowest median OD_570_ (0.105). Of the 55 pairwise comparisons, 16 were statistically significant, indicating moderate serotype-dependent divergence.

The classification of isolates into weak, moderate, and strong biofilm formers showed temperature- and serotype-specific patterns (Fig. 2B; Supplementary Table S6). At 25 °C, the majority of serotypes shifted toward stronger biofilm formation; notably, *S. Enteritidis* showed a marked increase in strong biofilm production (92.3%) compared to 46.2% at 15 °C. At 15 °C and 37 °C, most serotypes demonstrated weaker or more variable biofilm phenotypes, for example, *S. Choleraesuis* and *S. Stanley* formed exclusively weak biofilms at 37 °C. Violin-box plots stratified by serotype and temperature (Fig. 2C; Supplementary Table S7) further highlighted these temperature-modulated patterns. Principal component analysis (PCA; Fig. 2D; Supplementary Table S8) revealed serotype-characterized clustering. PC1 (39.0%) captured opposing biofilm formation at 25 °C and 37 °C, suggesting a temperature-driven trade-off, while PC2 (23.3%) reflected co-variation at 25 °C and 37 °C versus 15 °C, indicating cold-adaptive capacity. *S. Enteritidis* and *S. Typhimurium* localized to high PC1 regions, consistent with enhanced biofilm formation at moderate/warm temperatures. In contrast, *S. Agona* and *S. Goldcoast* clustered centrally, indicating generalist strategies, while *S. Typhi* and *S. Paratyphi* A showed lower PC2 values, suggesting enhanced cold-associated biofilm adaptation.

### 3.3 Antimicrobial resistance is not a major determinant of temperature-dependent biofilm formation

**Fig. 3:**
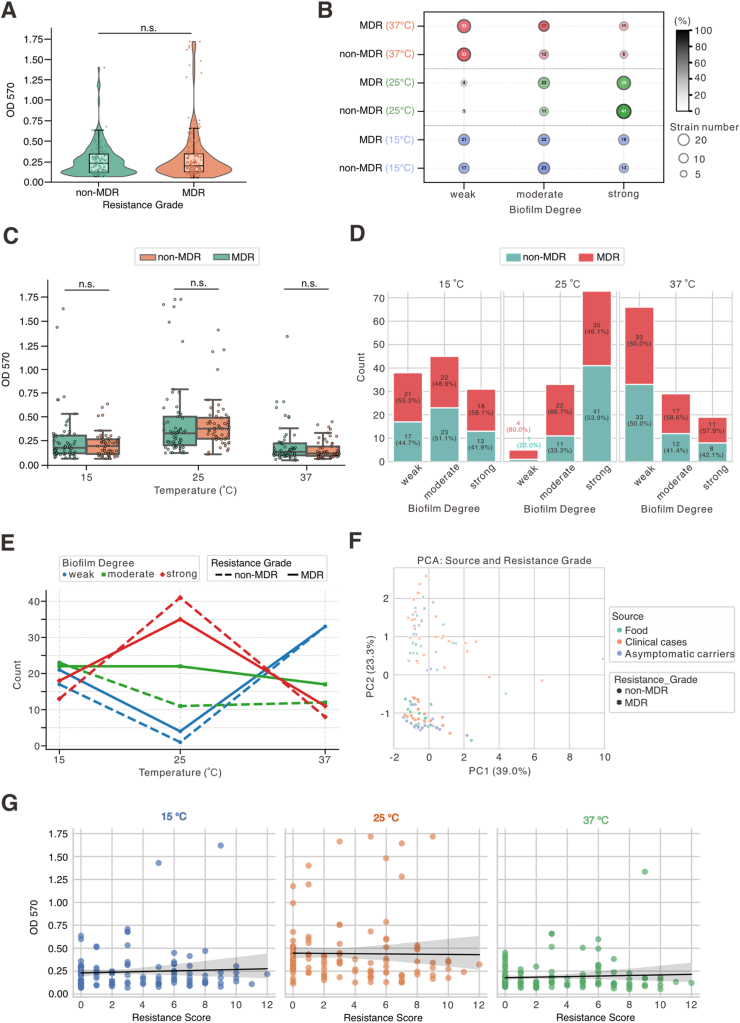
**Association between antimicrobial resistance and temperature-dependent biofilm formation in *Salmonella*. (A)** Violin-box plots of OD_570_ values in 114 *Salmonella* strains grouped by resistance grade (non-MDR *vs.* MDR). Individual data points are shown. No significant difference was detected between groups (Mann–Whitney *U* test, Bonferroni corrected, *p* = 0.7831). **(B)** Bubble plot showing the distribution of isolates by biofilm formation degree (weak, moderate, strong), resistance grade, and temperature (15 °C, 25 °C, 37 °C). Bubble size represents isolate count; color indicates temperature; grayscale reflects within-group percentage. Side colorbars denote absolute number and relative proportion. **(C)** Box plots with overlaid points illustrate OD_570_ distributions by resistance grade at each temperature. Statistical significance (Mann–Whitney *U* test) is indicated: *p* < 0.05, *p* < 0.01, *p* < 0.001; ns = not significant. MDR = blue; non-MDR = orange. **(D)** Stacked bar charts show the proportion of MDR and non-MDR isolates within each biofilm formation category at the three temperatures. Percentages are based on total isolates per temperature–biofilm group (see Supplementary Table S10). **(E)** Line plots depict the percentage of strains classified as weak, moderate, or strong biofilm formers across temperatures, stratified by resistance grade. Solid and dashed lines represent MDR and non-MDR groups, respectively. **(F)** PCA plot shows source- and resistance-dependent clustering. Strains are colored by isolation source (food = green, clinical = orange, healthy = blue) and shaped by resistance (MDR = crosses, non-MDR = dots). Food-derived strains cluster at PC1 < 0; MDR strains are enriched at PC1 > 2. **(G)** Pearson and Spearman correlation analyses between OD_570_ values and antimicrobial resistance scores across all temperatures. (For interpretation of the references to color in this figure legend, the reader is referred to the Web version of this article.)

To examine the relationship between biofilm formation and antimicrobial resistance, we compared OD_570_ values between multidrug-resistant (MDR) and non-MDR *Salmonella* strains. No significant difference was observed (Fig. 3A), indicating that resistance grade alone does not predict overall biofilm-forming capacity. When stratified by temperature, distinctions in biofilm phenotypes emerged across resistance groups (Fig. 3B and C; Supplementary Table S9–S10). At 25 °C, both MDR and non-MDR strains exhibited enhanced biofilm formation, with 77.4% of non-MDR and 57.4% of MDR strains classified as strong biofilm formers. In contrast, weak biofilm formation predominated at 37 °C, affecting 62.3% of non-MDR and 54.1% of MDR strains. At 15 °C, both groups showed balanced distributions across weak, moderate, and strong categories. These findings supported that biofilm formation is favored at 25 °C and suppressed at 37 °C, independent of resistance status. Consistently, neither MDR nor non-MDR status had a significant direct effect on biofilm formation, and no temperature-specific advantage was observed for MDR strains at any tested condition (Fig. 3C; Supplementary Table S10). MDR prevalence across biofilm strength categories did not align with increased biofilm capacity (Fig. 3D). At 25 °C, strong biofilm formers had the lowest MDR prevalence (46.1%), while weak formers had the highest (80.0%), further indicating that biofilm capacity does not drive MDR persistence. Line plot analysis (Fig. 3E; Supplementary Fig. S2) revealed similar temperature-dependent trajectories in both groups: biofilm strength peaked at 25 °C and declined at 37 °C, with more pronounced changes in non-MDR strains. This implies that thermal adaptation is independent of resistance phenotype.

PCA further separated resistance phenotypes (Fig. 3F; Supplementary Fig. S3; Supplementary Table S12), with clustering patterns reflecting thermally responsive biofilm behavior rather than inherent differences in capacity. Finally, correlation analyses confirmed the absence of association between biofilm formation and resistance score (Fig. 3G). Neither Pearson nor Spearman coefficients were significant across any source group or the full dataset (Supplementary Table S13). Together, these results demonstrate that biofilm formation in *Salmonella* is primarily temperature-driven and not enhanced by multidrug resistance.

### 3.4 Biofilm formation capacity varies by temperature and isolate source but shows no significant pairwise differences

**Fig. 4:**
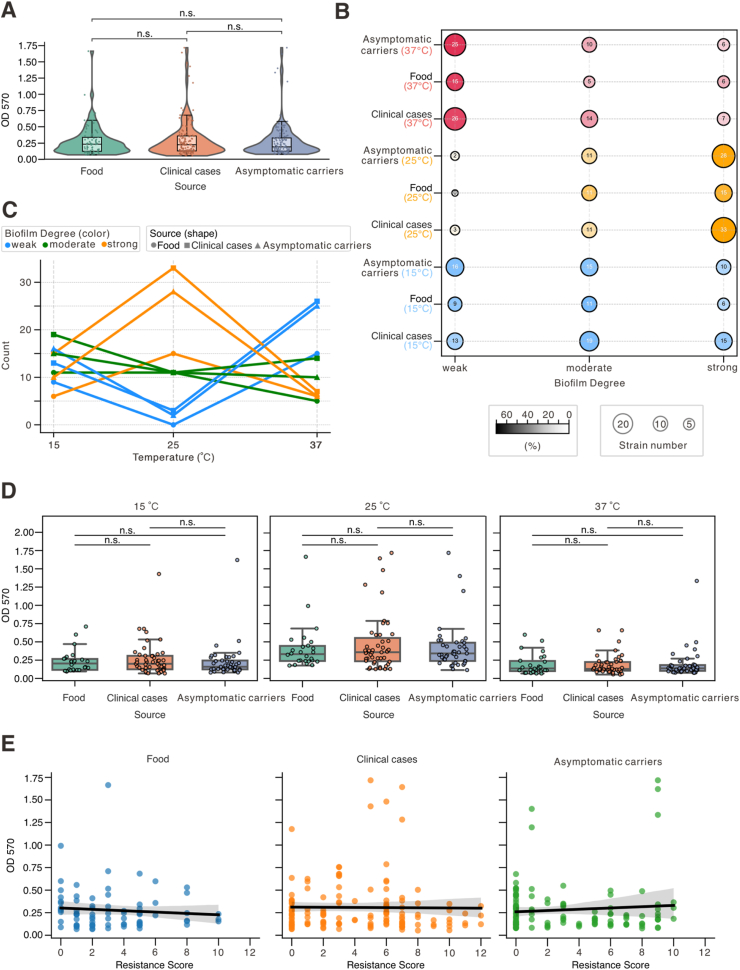
**Association between isolation source and temperature-dependent biofilm formation in *Salmonella*. (A)** Violin-box plots showing OD_570_ values for 114 *Salmonella* strains grouped by sources. Individual points are overlaid. No significant differences were found among sources (Mann–Whitney–Wilcoxon tests, Bonferroni corrected; all *p* = 1.000). **(B)** Bubble plot showing the distribution of isolates by biofilm-forming categories (weak, moderate, strong), source, and temperature (15 °C, 25 °C, 37 °C). Bubble size indicates isolate count; color represents temperature; grayscale reflects the within-group percentage. **(C)** Line plots illustrating the proportion of isolates in each biofilm-forming category across temperatures, stratified by source. Shapes represent sources (circle = Food, square = Clinical cases, triangle = Asymptomatic carriers); colors denote biofilm strength (blue = weak, green = moderate, orange = strong). **(D)** Box plots with overlaid scatter points show OD_570_ values by source at each temperature. Boxes indicate interquartile range, medians, and 1.5 × IQR whiskers. Statistical comparisons between sources were performed using Mann–Whitney U tests with Bonferroni correction (*p* < 0.05, *p* < 0.01, *p* < 0.001; ns = not significant). Colors: Food (green), Clinical cases (orange), Asymptomatic carriers (blue). **(E)** Pearson and Spearman correlation analyses between OD_570_ values and source categories across all temperatures. (For interpretation of the references to color in this figure legend, the reader is referred to the Web version of this article.)

As shown in Fig. 4A, under the tested conditions and given the current sample size, no statistically significant difference in biofilm formation capacity (OD_570_) was observed among the broadly defined source categories (food, clinical, asymptomatic carriers). Further stratification by temperature and source revealed distinct patterns in biofilm phenotype distribution (Fig. 4B and C; Supplementary Fig. S4; Supplementary Table S11–S12). At 25 °C, all three source groups exhibited increased proportions of strong biofilm-forming strains, with clinical isolates reaching the highest proportion (70.2%). In contrast, at 37 °C, weak biofilm formation became dominant, particularly among clinical (55.3%) and asymptomatic carriers (61.0%) isolates. At 15 °C, a more balanced distribution across weak, moderate, and strong biofilm-forming categories was observed. OD_570_ distributions across temperatures for each source are shown in Fig. 4D and Supplementary Table S14. Although numerical differences were noted, such as slightly stronger biofilm formation in food isolates at 15 °C and in clinical strains at 25 °C, none of these differences reached statistical significance. This reinforces the conclusion that temperature is a dominant factor in shaping biofilm phenotypes, whereas source-related differences are relatively modest and statistically non-significant. Correlation analyses between biofilm formation (OD_570_) and antimicrobial resistance score showed no significant associations within any source group or in the overall dataset (Fig. 4E; Supplementary Table S15).

### 3.5 Serotype and temperature are the primary determinants of biofilm formation

**Fig. 5:**
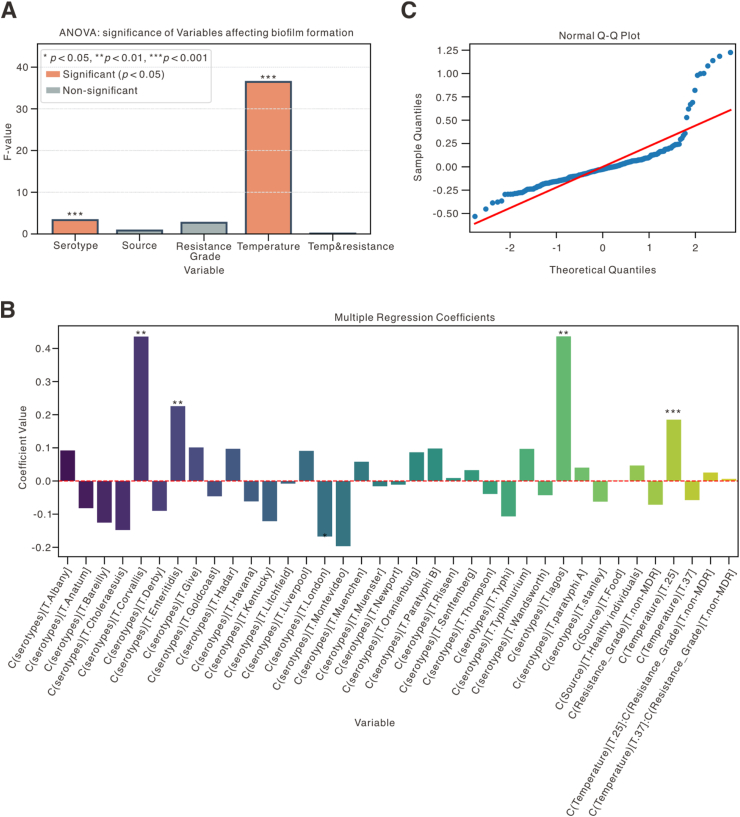
**Multivariate regression analysis of factors influencing temperature-dependent biofilm formation in *Salmonella*. (A)** ANOVA significance analysis demonstrating main effects. Bars represent F-statistics for each variable. Red bars indicate statistically significant predictors (*p* < 0.05), gray bars denote non-significant ones. Serotype (F = 3.31, df = 30, *p* = 6.91 × 10^−8^) and temperature (F = 36.46, df = 2, *p* = 6.40 × 10^−15^) are significant contributors to biofilm variation. Source (*p* = 0.442), resistance grade (*p* = 0.103), and their interaction with temperature (*p* = 0.913) show no significant effects. **(B)** Bar plot of regression coefficients for model predictors (primarily serotype dummy variables). Bars above and below the baseline represent positive and negative effects on OD570, respectively. Asterisks indicate significance: *p* < 0.05 (∗), *p* < 0.01 (∗∗), *p* < 0.001 (∗∗∗). **(C)** Scatter plot (Quantile-Quantile plot) assessing the normality of residuals from the regression model. Data points closely following the red reference line indicate adherence to a normal distribution. (For interpretation of the references to color in this figure legend, the reader is referred to the Web version of this article.)

To further validate the drivers of biofilm formation, we performed ANOVA and multiple regression analyses (Fig. 5; Supplementary Fig. S5; Supplementary Table S16). The results confirmed that serotype (F = 3.31, *p* = 6.91 × 10^−8^) and temperature (F = 36.46, *p* = 6.40 × 10^−15^) exert significant and independent effects on OD_570_ values, indicating their dominant roles in modulating biofilm-forming capacity. Corresponding effect size estimates (partial η^2^) of 0.25 and 0.19 suggest moderate biological relevance (Fig. 5A; Supplementary Table S16). In contrast, bacterial source (*p* = 0.442), resistance grade (*p* = 0.103), and the temperature × resistance grade interaction (*p* = 0.913) were not significant (Supplementary Table S16), supporting that resistance and source do not contribute substantially to temperature-dependent biofilm phenotypes. Regression coefficient analysis (Fig. 5B; Supplementary Fig. S6) revealed that specific serotypes, notably *S. Corvallis*, had a strong positive effect on biofilm formation at 25 °C (regression coefficient = 0.9; *p* < 0.01). Other serotype variables exhibited variable effects, most of which were non-significant. These findings reinforce earlier observations that strain-level genetic background, particularly serotype identity, may modulate biofilm potential under thermal conditions. In particular, the Q–Q plot of residuals (Fig. 5C) indicated approximate normality in central quantiles, supporting the appropriateness of the regression model, despite minor tail deviations. Levene's test confirmed homogeneity of variance (*p* > 0.05), validating the model's assumptions. Together, these analyses provide robust statistical support for the conclusion that biofilm formation in *Salmonella* is significantly shaped by temperature and serotype, but not by source or resistance profile.

### 3.6 Presence of biofilm-associated genes does not predict biofilm-forming capacity

PCR-based screening of 28 known biofilm-associated genes across the 114 *Salmonella* isolates revealed that 23 genes were universally present, regardless of biofilm-forming phenotype (Table 2). This widespread presence suggests that core biofilm-related genetic elements are conserved among diverse *Salmonella* strains, independent of their observed biofilm strength. Notably, only five genes (*rcK*, *fimH*, *steB*, pef*A*,and *pefB*) exhibited variable distribution. The *rcK* gene was detected exclusively in a few moderate or strong biofilm producers, all belonging to *S. Enteritidis*, suggesting a potential serotype-specific association rather than a direct correlation with biofilm intensity. Conversely, a small subset of weak or non-biofilm-producing strains, all identified as *S. Choleraesuis* (belongs to the weak biofilm category), lacked the *flmH* gene, again implying serotype-linked variation rather than functional absence in biofilm producers. The *pefA* and *pefB* genes were detected in 19.3% of the isolates (22/114), and were found across all biofilm-forming categories, including weak and non-producers. Additionally, the *steB* gene was absent in some strains that nonetheless exhibited clear biofilm formation, indicating that its presence is not essential for biofilm phenotype expression under the tested conditions.

**Table 2:** Biofilm-related gene distribution in bacterial isolates with varying biofilm formation abilities at 37 °C (%).

Genes	Non-biofilm producer (n = 6)	Week biofilm producers (n = 60)	Moderate biofilm producers (n = 29)	Strong biofilm producers (n = 19)
*adrA*	100	100	100	100
*bcsA*	100	100	100	100
*csgB*	100	100	100	100
*csgD*	100	100	100	100
*csrA*	100	100	100	100
*csrB*	100	100	100	100
*fliC*	100	100	100	100
*fimH*	50(3/6)	98.33(59/60)	100	100
*glyA*	100	100	100	100
*igaA*	100	100	100	100
*invA*	100	100	100	100
*lpfA*	100	100	100	100
*luxS*	100	100	100	100
*misL*	100	100	100	100
*mlrA*	100	100	100	100
*ompR*	100	100	100	100
*pefA*	66.67(4/6)	6.67(4/60)	17.24(5/29)	47.37(9/19)
*pefB*	66.67(4/6)	6.67(4/60)	17.24(5/29)	47.37(9/19)
*pfs*	100	100	100	100
*rcK*	0	0	17.24(5/29)	36.84(7/19)
*rpoS*	100	100	100	100
*sdiA*	100	100	100	100
*sipB*	100	100	100	100
*sipC*	100	100	100	100
*sirA*	100	100	100	100
*srgA*	100	100	100	100
*steB*	100	63.33(38/60)	75.86(22/29)	78.95(15/19)
*wcaA*	100	100	100	100

## 4 Discussion

*Salmonella* remains one of the most significant foodborne pathogens globally, responsible for a substantial burden of illness and economic losses every year. Producing biofilms is a critical survival mechanism that enhances *Salmonella* persistence, resistance, and transmission in various environments. The ability of *Salmonella* to form biofilms is highly variable and influenced by serotypes, genetic factors, and environmental conditions [26].

The optimal growth temperature for *Salmonella* is around 37 °C, coinciding with the body temperature of its human and warm-blooded hosts. However, the temperature required for robust biofilm formation appears to diverge toward lower temperatures. For instance, several studies have reported enhanced biofilm formation at lower temperatures: 25 °C was identified as optimal compared to 37 °C [27,28], 28 °C was favourable over 37 °C [29], and 22 °C supported greater biofilm production than 35 °C[30]. Similarly, biofilm formation was found to be stronger at 25 °C than at 15 °C [31]. Given the close association between biofilm formation and bacterial tolerance to antimicrobials and environmental stresses, and the documented temperature-dependent nature of this phenotype, the present study aimed to assess the influence of temperature on the biofilm-forming capacity of 114 *Salmonella* isolates [1]. In line with these findings, the results of the present study demonstrated that the biofilm-forming capacity of *Salmonella* isolates was significantly stronger at 25 °C than at either 37 °C or 15 °C, which was consistent with previous reports [32,33]. This temperature-dependent pattern occurred despite planktonic growth rates being highest at 37 °C, as revealed by analysis of representative strains, indicating a clear decoupling between optimal growth and optimal biofilm formation temperatures (Supplementary Fig. S1). Curli are important for biofilm development in *Salmonella* [34,35]. Notably, for most *Salmonella* strains, curli expression is highest at temperatures below 30 °C, which may contribute to the enhanced biofilm formation observed at this range of temperature. Beyond the regulation of proteinaceous fibers, temperature reduction exerts a broader influence on biofilm architecture by remodeling the EPS matrix [36]. In contrast, under host-associated temperatures, *Salmonella* prioritize virulence factor expression for invasion, typically inhibiting biofilm formation [37,38]. This reflects a resource allocation trade-off between survival and pathogenicity.

*Salmonella* serotype is closely associated with its source. For instance, serovars *S.Typhi* and *S. Paratyphi A* primarily infect humans. *S. Choleraesuis* predominantly colonizes pigs and rarely infects humans. Similarly, *S. Dublin* shows a strong host preference for cattle, while *S. Heidelberg* is closely linked to poultry[39]. *Salmonella* serotype significantly influences biofilm formation, attracting increasing research attention [25]. In this study, the 31 analyzed *Salmonella* serotypes included 15 serotypes prevalent in China (Typhimurium, Enteritidis, Derby, Agona, Indiana, Anatum, Senftenberg, Stanley, London, Thompson, Rissen, Newport, Choleraesuis, Corvallis, and Kentucky) and 16 rare serotypes [40]. Among them, *S. Enteritidis* and *S. Typhimurium,* mainly isolated from human samples, exhibited the highest biofilm-forming capacity across all tested temperatures (15 °C, 25 °C, and 37 °C). In contrast, *S. Derb*y, as a major epidemic serotype, showed a comparatively weak biofilm-forming ability, which was consistent with the findings reported by Wang et al. [41]. Notably, we identified a strain of *S. Typhimurium* (designated S100) that demonstrated the strongest biofilm formation at all three temperatures, suggesting enhanced colonization capacity and pathogenic potential in both natural environments and host organisms. Comparisons with other studies highlight context-dependent differences. *S*. *Enteritidis* showed the greatest biofilm formation in two studies of meat and meat contact surfaces [42,43]. Díez-García et al. identified *S. Agona* and *S. Typhi* as the strongest biofilm formers under their specific conditions (37 °C, 24 h), although this conclusion was based on a limited number of strains (3 for *S. Agona* and 1 for *S. Typhi*) [44]. Similarly, Silva et al. reported that *S. Gallinarum* and *S. Minnesota* formed stronger biofilms than *S. Enteritidis* and *S. Typhimurium* in a study of six poultry-derived serotypes, though the number of strains per serotype was not specified [45]. In contrast, Locke et al. reported that among 81 *Salmonella* isolates from clinically ill livestock incubated at 37 °C for 48 h, *S. Heidelberg and S. Enteritidis* exhibited the strongest biofilm formation [46]. The variation in biofilm-forming abilities among different *Salmonella* serotypes can be attributed to a combination of genetic, structural, regulatory, and environmental factors. Furthermore, *Salmonella* serotype is associated with MDR. The most frequently reported MDR serotypes were Typhimurium, Enteritidis, Newport, and Heidelberg [47]. In our previous study, common serotypes (e.g., Enteritidis, Typhimurium, Derby) demonstrated a significantly higher prevalence of MDR compared to rare serotypes[24]. Collectively, these findings demonstrate that *Salmonella* serotypes exhibit distinct, temperature-modulated biofilm phenotypes, supporting the concept of serotype-specific thermal adaptation that may play a role in niche survival and transmission dynamics.

Biofilms serve as a protective mechanism that enhances bacterial tolerance to antibiotic stress. However, the relationship between biofilm formation and antibiotic resistance remains controversial. In *Escherichia coli*, most studies indicate a positive correlation between biofilm formation and antibiotic resistance, although some report no significant association[48]. In contrast, *Acinetobacter baumannii* exhibits a statistically significant negative correlation between antibiotic resistance and biofilm-forming capacity [49]. For *Salmonella*, Márquez et al. [50] demonstrated no association between biofilm-forming capacity and antimicrobial susceptibility in planktonic strains, contrasting with Siddique et al.[51] who identified a significant positive correlation between these phenotypic traits. In the present study, we observed no significant correlation between *Salmonella* biofilm formation capacity and planktonic bacterial drug resistance at any of the three cultivation temperatures (15 °C, 25 °C, and 37 °C). Therefore, our findings support the notion that biofilm-forming capacity is independent of resistance level. Notably, our results revealed that 21, 32, and 11 strains exhibited robust biofilm-forming capacity coupled with MDR phenotypes at the three temperatures, respectively. These dual-risk strains demonstrate enhanced transmission potential and therapeutic resistance, posing significant challenges for clinical management and infection control.

Isolation source is regarded as an environmental factor that may affect biofilm formation [52,53]. Clinical isolates tend to be more adapted to antibiotics and the host immune system, while food-derived strains are more adaptable to environments such as low temperatures and high-salt conditions [52,53]. Our results indicate that biofilm formation ability does not differ significantly among strains from food, clinical, and healthy carrier sources at the three tested temperatures, consistent with previous findings from Liu et al. [25]. A possible explanation for this observation is that the *Salmonella* strains circulating among these reservoirs are not genetically distinct. This facilitates a continuous transmission cycle: contaminated food infects human hosts, and infected individuals shed the pathogen in feces, leading to subsequent food contamination. Biofilms play an important role in maintaining *Salmonella*'s chronic infection state [9,54], which may further support its persistence and dissemination within this cycle. Whether an infection presents as symptomatic disease or remains asymptomatic depends primarily on the host's immune status, and these clinical states can interconvert as immune status changes. Moreover, this study was constrained by its limited sample size and low representation for many serovars (<3 isolates). The within-serovar variation also remains unclear. We note that the food category includes diverse subtypes (e.g., vegetables, spices, carcass-derived products), and some serovars are strongly associated with specific sources (e.g., *S. Choleraesuis* with swine). These factors may affect biofilm phenotypes and should be addressed in future studies with larger and more stratified sample sets. Therefore, our analysis was limited to comparing biofilm formation ability across broadly defined source categories, which may have influenced the findings.

The ability of bacteria to form biofilms is determined by genotype, but is also influenced by factors such as gene expression regulation, gene mutations, environmental factors, and post-translational modifications. This study evaluated 28 genes associated with *Salmonella* biofilm formation, with only *rck*, *fimH*, *steB*, and *pefA* showing significant differences. In contrast, using whole-genome sequencing, Locke et al. reported a different set of biofilm-associated genes, including *csrA*, *csrB*, *pefA*, *pfs*, *rck*, *srgA*, and *yjfO* [46]. The *rck* operon regulates the expression of plasmid-encoded fimbriae, thereby affecting biofilm formation. We found that the *rck* gene was unique to *S. Enteritidis*, whereas Yin et al. reported it to be specific to *S. Typhimurium* [28]. The *fimH* gene encodes the adhesin subunit of mannose-specific type 1 fimbriae [55]. We observed that *fimH* gene deficiency occurred exclusively in *S. Choleraesuis* strains, which exhibited either no biofilm formation or only weak biofilm formation at 37 °C. This suggests that host adaptation may influence the carriage of biofilm-forming genes. The *pefA*-encoded major fimbrial subunit mediates *Salmonella* colonization and biofilm formation [56]. This gene was exclusively detected in *Salmonella* enterica serovars Enteritidis, Typhimurium, Choleraesuis, Senftenberg, Typhi, and Lagos in our study. Together, these findings suggest that the mere presence of biofilm-related genes does not directly translate to enhanced biofilm formation. This aligns with earlier observations that biofilm capacity is shaped more by serotype background and environmental factors such as temperature, rather than by the differential presence or absence of canonical biofilm genes. The presence of genes only indicates genetic potential, whereas dynamic regulation at the transcriptional and translational levels is the direct driver of differences in biofilm-forming phenotypes [57]. For instance, transcription of *BrfS*, a novel MlrA-like regulator in *Salmonella*, increases at 20 °C under nutrient limitation, thereby promoting biofilm formation [36]. Similarly, in *E. coli* K-12, multiple biofilm-related genes such as *adrA, csgA*, and *mlrA* are upregulated at 23 °C compared to 37 °C, further supporting the role of temperature in modulating biofilm development [58]. Future studies would benefit from approaches such as transcriptomics to investigate the expression variations of the same genes across strains from different sources, thereby more precisely revealing the adaptive mechanisms underlying biofilm formation.

This study has some limitations. First, biofilm formation was assessed on abiotic polystyrene surfaces in nutrient-rich medium, which may not fully replicate the complex conditions on host tissues or food-contact surfaces. Second, biofilm formation is a dynamic process, and evaluating it at a single time point (3 days) does not capture its temporal development. We acknowledge that extending the incubation to later time points (e.g., 5 or 7 days) to investigate long-term biofilm maturation under different temperatures would be a valuable direction for future studies. Third, the analyzed *Salmonella* strains were predominantly from Hubei Province, China, with a limited sample size. Future studies should include broader geographical sampling to support more generalizable conclusions. Lastly, this study assessed the biofilm-forming ability of *Salmonella* cultivated on abiotic polystyrene surfaces in nutrient-rich TSB medium, which may not fully simulate the complex physicochemical and biological conditions encountered in host tissues or on food-processing contact surfaces. In addition, crystal violet staining quantifies total biomass and cannot differentiate between live cells and EPS matrix, limiting interpretation of biofilm viability and structural composition. Future studies using complementary approaches such as live/dead staining, confocal microscopy, or flow-cell systems under more realistic environmental conditions would provide deeper insights into *Salmonella* biofilm physiology.

In conclusion, this study demonstrate that biofilm formation in *Salmonella* is significantly influenced by temperature, with optimal biofilm production occurring at 25 °C. Furthermore, biofilm-forming capacity varies among serotypes, with *S. Enteritidis*, *S. Thompson*, and *S. Typhimurium* showing the strongest biofilm formation. However, no direct association was found between biofilm production and antimicrobial resistance or strain origin, suggesting that these traits may be independently regulated. These results contribute to a better understanding of *Salmonella* biofilm dynamics and highlight the need for further research to elucidate the mechanisms linking biofilm formation, virulence, and antimicrobial resistance in this important foodborne pathogen.

## CRediT authorship contribution statement

**Jun Lv:** Software, Project administration. **Tingting Ju:** Methodology, Formal analysis. **Wenlin Ye:** Validation, Formal analysis. **Changlin Yu:** Software, Resources. **Qi Cheng:** Project administration, Investigation, Conceptualization. **Haitang Xiong:** Resources, Methodology. **Xinyun Fan:** Validation, Software. **Lanfang Liu:** Software, Methodology. **Zhiyong Song:** Writing – original draft, Resources. **Bin Luo:** Software, Resources. **Yonghong Zhang:** Validation, Formal analysis.

## Consent to publish

Not applicable.

## Ethics approval

This study received ethical approval by the Science and Technology Ethics Committee of Hubei University of Medicine (2024-PR-097).

## Declaration of generative AI and AI-assisted technologies in the writing process

During the preparation of this work the author(s) used [DeepSeek] (https://www.deepseek.com) for language refinement. After using this tool/service, the author(s) reviewed and edited the content as needed and take(s) full responsibility for the content of the publication.

## Funding

This work was supported by the Science Research Program of Hubei Provincial Department of Education (T2023016); the Hubei Provincial Natural Science Foundation of China (2025BBB028), the Joint supported by Hubei Provincial Natural Science Foundation and Shiyan-of China (2025AFD179, 2025AFD194); the Advantages Discipline Group (Biology and Medicine) Project in Higher Education of Hubei Province (2021-2025) (2022BMXKQT4), and the Taihe Hospital Research Foundation (2024JJXM048).

## Conflicts of interest

The authors declare they have no competing interests.

## Data Availability

Data will be made available on request.

## References

[1] Aleksandrowicz A., Carolak E., Dutkiewicz A., Błachut A., Waszczuk W., Grzymajlo K. (2023). Better together-Salmonella biofilm-associated antibiotic resistance. Gut Microbes.

[2] Pino M., Mujica K., Mora-Uribe P., Garcias-Papayani H., Paillavil B., Avendaño C., Flores-Crisosto D., Norambuena R., Rojas-Martínez V., Aguilera M., Muñoz N.C., Cifuentes P., Pieringer H., Ulloa S. (2025). Research Note: reduction of Salmonella load in Brazilian commercial chicken farms using INSPEKTOR®: a bacteriophage-based product. Poult Sci.

[3] Lamichhane B., Mawad A.M.M., Saleh M., Kelley W.G., Harrington P.J., Lovestad C.W., Amezcua J., Sarhan M.M., El Zowalaty M.E., Ramadan H., Morgan M., Helmy Y.A. (2024). Salmonellosis: an overview of epidemiology, pathogenesis, and innovative approaches to mitigate the antimicrobial resistant infections. Antibiotics (Basel).

[4] Qamar F.N., Hussain W., Qureshi S. (2022). Salmonellosis including enteric fever. Pediatr Clin North Am.

[5] Zha L., Garrett S., Sun J. (2019). Salmonella infection in chronic inflammation and gastrointestinal cancer. Diseases.

[6] Paudyal N., Pan H., Wu B., Zhou X., Zhou X., Chai W., Wu Q., Li S., Li F., Gu G., Wang H., Hu Q., Xu X., Li Y., Yue M. (2020). Persistent asymptomatic human infections by Salmonella enterica Serovar Newport in China. mSphere.

[7] Näsström E., Jonsson P., Johansson A., Dongol S., Karkey A., Basnyat B., Tran Vu Thieu N., Trinh Van T., Thwaites G.E., Antti H., Baker S. (2018). Diagnostic metabolite biomarkers of chronic typhoid carriage. PLoS Negl Trop Dis.

[8] Ruby T., McLaughlin L., Gopinath S., Monack D. (2012). Salmonella's long-term relationship with its host. FEMS Microbiol Rev.

[9] González J.F., Hitt R., Laipply B., Gunn J.S. (2022). The effect of the gallbladder environment during chronic infection on salmonella persister cell Formation. Microorganisms.

[10] Jahan F., Chinni S.V., Samuggam S., Reddy L.V., Solayappan M., Su Yin L. (2022). The complex mechanism of the Salmonella typhi biofilm Formation that facilitates pathogenicity: a review. Int J Mol Sci.

[11] Lu X., Luo M., Wang M., Zhou Z., Xu J., Li Z., Peng Y., Zhang Y., Ding F., Jiang D., Zhou C., Yang L., Zhao W., Ma T., Pang B., Yan M., Wu Y., Wu Y., Kan B. (2024). High carriage and possible hidden spread of multidrug-resistant Salmonella among asymptomatic workers in Yulin, China. Nat Commun.

[12] Woh P.Y., Thong K.L., Behnke J.M., Lewis J.W., Zain S.N.M. (2017). Characterization of nontyphoidal salmonella isolates from asymptomatic migrant food handlers in peninsular Malaysia. J Food Protect.

[13] Costa R.C., Bertolini M., Costa Oliveira B.E., Nagay B.E., Dini C., Benso B., Klein M.I., Barāo V.A.R., Souza J.G.S. (2023). Polymicrobial biofilms related to dental implant diseases: unravelling the critical role of extracellular biofilm matrix. Crit Rev Microbiol.

[14] Devaraj A., González J.F., Eichar B., Thilliez G., Kingsley R.A., Baker S., Allard M.W., Bakaletz L.O., Gunn J.S., Goodman S.D. (2021). Enhanced biofilm and extracellular matrix production by chronic carriage versus acute isolates of Salmonella Typhi. PLoS Pathog.

[15] Jiang Y., Geng M., Bai L. (2020). Targeting biofilms therapy: current research strategies and development hurdles. Microorganisms.

[16] Mishra A., Tabassum N., Aggarwal A., Kim Y.M., Khan F. (2024). Artificial intelligence-driven analysis of antimicrobial-resistant and biofilm-forming pathogens on biotic and abiotic surfaces. Antibiotics (Basel).

[17] Sokaribo A.S., Hansen E.G., McCarthy M., Desin T.S., Waldner L.L., MacKenzie K.D., Mutwiri G., Herman N.J., Herman D.J., Wang Y., White A.P. (2020). Metabolic activation of CsgD in the regulation of salmonella biofilms. Microorganisms.

[18] Hahn M.M., González J.F., Gunn J.S. (2021). Salmonella biofilms tolerate hydrogen peroxide by a combination of extracellular polymeric substance barrier function and catalase enzymes. Front Cell Infect Microbiol.

[19] Papavasileiou K., Papavasileiou E., Tseleni-Kotsovili A., Bersimis S., Nicolaou C., Ioannidis A., Chatzipanagiotou S. (2010). Comparative antimicrobial susceptibility of biofilm versus planktonic forms of Salmonella enterica strains isolated from children with gastroenteritis. Eur J Clin Microbiol Infect Dis.

[20] Stewart P.S., Costerton J.W. (2001). Antibiotic resistance of bacteria in biofilms. Lancet.

[21] Watters C., Fleming D., Bishop D., Rumbaugh K.P. (2016). Host responses to biofilm. Prog Mol Biol Transl Sci.

[22] Moshiri J., Kaur D., Hambira C.M., Sandala J.L., Koopman J.A., Fuchs J.R., Gunn J.S. (2018). Identification of a small molecule anti-biofilm agent against Salmonella enterica. Front Microbiol.

[23] Goller C.C., Romeo T. (2008). Environmental influences on biofilm development. Curr Top Microbiol Immunol.

[24] Lv J., Geng L., Ye W., Gong S., Wu J., Ju T., Li L., Liu L., Zhang Y. (2024). Antimicrobial resistance and genetic relatedness of Salmonella serotypes isolated from food, asymptomatic carriers, and clinical cases in Shiyan, China. PLoS One.

[25] Liu X., Jiang Z., Liu Z., Li D., Liu Z., Dong X., Yan S., Zhu L., Cui D., Chen L., Wang J. (2023). Biofilm-forming ability of Salmonella enterica strains of different serotypes isolated from multiple sources in China. Microb Pathog.

[26] Harrell J.E., Hahn M.M., D'Souza S.J., Vasicek E.M., Sandala J.L., Gunn J.S., McLachlan J.B. (2020). Salmonella biofilm Formation, chronic infection, and immunity within the intestine and hepatobiliary tract. Front Cell Infect Microbiol.

[27] Akinola S.A., Tshimpamba M.E., Mwanza M., Ateba C.N. (2020). Biofilm production potential of salmonella serovars isolated from chickens in North West Province, South Africa. Pol J Microbiol.

[28] Yin B., Zhu L., Zhang Y., Dong P., Mao Y., Liang R., Niu L., Luo X. (2018). The characterization of biofilm Formation and detection of biofilm-related genes in salmonella isolated from beef processing plants. Foodb Pathog Dis.

[29] Ćwiek K., Korzekwa K., Tabiś A., Bania J., Bugla-Płoskońska G., Wieliczko A. (2020). Antimicrobial resistance and biofilm Formation capacity of Salmonella enterica Serovar Enteritidis strains isolated from poultry and humans in Poland. Pathogens.

[30] Piras F., Fois F., Consolati S.G., Mazza R., Mazzette R. (2015). Influence of temperature, source, and serotype on biofilm Formation of Salmonella enterica isolates from pig slaughterhouses. J Food Protect.

[31] Obe T., Richards A.K., Shariat N.W. (2022). Differences in biofilm formation of Salmonella serovars on two surfaces under two temperature conditions. J Appl Microbiol.

[32] Bashir A., Azeem A., Stedman Y., Hilton A.C. (2019). Pet food factory isolates of salmonella serotypes do not demonstrate enhanced biofilm Formation compared to serotype-matched clinical and veterinary isolates. BioMed Res Int.

[33] Lianou A., Koutsoumanis K.P. (2012). Strain variability of the biofilm-forming ability of Salmonella enterica under various environmental conditions. Int J Food Microbiol.

[34] Barnhart M.M., Chapman M.R. (2006). Curli biogenesis and function. Annu Rev Microbiol.

[35] Nicastro L.K., Tursi S.A., Le L.S., Miller A.L., Efimov A., Buttaro B., Tam V., Tukel C. (2019). Cytotoxic Curli intermediates form during Salmonella biofilm development. J Bacteriol.

[36] Tulin G., Méndez A.A.E., Figueroa N.R., Smith C., Folmer M.P., Serra D., Wade J.T., Checa S.K., Soncini F.C. (2025). Integration of BrfS into the biofilm-controlling cascade promotes sessile Salmonella growth at low temperatures. Biofilms.

[37] Roncarati D., Vannini A., Scarlato V. (2025). Temperature sensing and virulence regulation in pathogenic bacteria. Trends Microbiol.

[38] Roy P.K., Ha A.J., Mizan M.F.R., Hossain M.I., Ashrafudoulla M., Toushik S.H., Nahar S., Kim Y.K., Ha S.D. (2021). Effects of environmental conditions (temperature, pH, and glucose) on biofilm formation of Salmonella enterica serotype Kentucky and virulence gene expression. Poult Sci.

[39] Gal-Mor O. (2019). Persistent infection and long-term carriage of typhoidal and nontyphoidal salmonellae. Clin Microbiol Rev.

[40] Chen J., Huang L., An H., Wang Z., Kang X., Yin R., Jia C., Jin X., Yue M. (2024). One Health approach probes zoonotic non-typhoidal Salmonella infections in China: a systematic review and meta-analysis. J Glob Health.

[41] Wang H., Ding S., Dong Y., Ye K., Xu X., Zhou G. (2013). Biofilm formation of Salmonella serotypes in simulated meat processing environments and its relationship to cell characteristics. J Food Protect.

[42] Counihan K.L., Tilman S., Uknalis J., Mukhopadhyay S., Niemira B.A., Bermudez-Aguirre D. (2025). Attachment and biofilm Formation of eight different salmonella serotypes on three food-contact surfaces at different temperatures. Microorganisms.

[43] Manafi L., Aliakbarlu J., Dastmalchi Saei H. (2020). Antibiotic resistance and biofilm formation ability of Salmonella serotypes isolated from beef, mutton, and meat contact surfaces at retail. J Food Sci.

[44] Díez-García M., Capita R., Alonso-Calleja C. (2012). Influence of serotype on the growth kinetics and the ability to form biofilms of Salmonella isolates from poultry. Food Microbiol.

[45] Silva P., Goulart L.R., Reis T.F.M., Mendonça E.P., Melo R.T., Penha V.A.S., Peres P., Hoepers P.G., Beletti M.E., Fonseca B.B. (2019). Biofilm Formation in different salmonella serotypes isolated from poultry. Curr Microbiol.

[46] Locke S.R., Vinayamohan P.G., Diaz-Campos D., Habing G. (2025). Biofilm-forming abilities of salmonella serovars isolated from clinically ill livestock at 48 and 168 h. J Food Protect.

[47] Parisi A., Crump J.A., Glass K., Howden B.P., Furuya-Kanamori L., Vilkins S., Gray D.J., Kirk M.D. (2018). Health outcomes from multidrug-resistant salmonella infections in high-income countries: a systematic review and meta-analysis. Foodb Pathog Dis.

[48] Garousi M., Monazami Tabar S., Mirazi H., Asgari P., Sabeghi P., Salehi A., Khaledi A., Ghenaat Pisheh Sanani M., Mirzahosseini H.K. (2022). A global systematic review and meta-analysis on correlation between biofilm producers and non-biofilm producers with antibiotic resistance in Uropathogenic Escherichiacoli. Microb Pathog.

[49] Qi L., Li H., Zhang C., Liang B., Li J., Wang L., Du X., Liu X., Qiu S., Song H. (2016). Relationship between antibiotic resistance, biofilm Formation, and biofilm-specific resistance in Acinetobacter baumannii. Front Microbiol.

[50] Márquez M.L.F., Burgos M.J.G., Pulido R.P., Gálvez A., López R.L. (2018). Correlations among resistances to different antimicrobial compounds in salmonella strains from Hen eggshells. J Food Protect.

[51] Siddique A., Azim S., Ali A., Andleeb S., Ahsan A., Imran M., Rahman A. (2021). Antimicrobial resistance profiling of biofilm forming non typhoidal Salmonella enterica isolates from poultry and its associated food products from Pakistan. Antibiotics (Basel).

[52] Lakicevic B.Z., Den Besten H.M.W., De Biase D. (2021). Landscape of stress response and virulence genes among Listeria monocytogenes strains. Front Microbiol.

[53] Sibale L.L., Lo S.W., Kalata N., Nyazika T.K., Mitole N., Dyster V., Kusakala A., Khwiya M., Sagawa G., Phiri J.A., Kalizang'oma A., Swarthout T.D., Malisita K., Kamng'ona A.W., Heyderman R.S., Bentley S.D., Kwambana-Adams B.A., Chaguza C., Jambo K.C. (2025). Within-host genetic diversity of pneumococcal serotype 3 during one-year prolonged carriage in a healthy adult. Nat Commun.

[54] Białucha A., Gospodarek-Komkowska E., Kwiecińska-Piróg J., Skowron K. (2020). Influence of selected factors on biofilm Formation by Salmonella enterica strains. Microorganisms.

[55] Cohen E., Azriel S., Auster O., Gal A., Zitronblat C., Mikhlin S., Scharte F., Hensel M., Rahav G., Gal-Mor O. (2021). Pathoadaptation of the passerine-associated Salmonella enterica serovar Typhimurium lineage to the avian host. PLoS Pathog.

[56] Alshalchi S., Hayer S.S., An R., Munoz-Aguayo J., Flores-Figueroa C., Nguyen R., Lauer D., Olsen K., Alvarez J., Boxrud D., Cardona C., Vidovic S. (2017). The possible influence of non-synonymous point mutations within the FimA adhesin of non-typhoidal salmonella (NTS) isolates in the process of host adaptation. Front Microbiol.

[57] Katharios-Lanwermeyer S., O'Toole G.A. (2022). Biofilm maintenance as an active process: evidence that biofilms work hard to stay put. J Bacteriol.

[58] White-Ziegler C.A., Um S., Pérez N.M., Berns A.L., Malhowski A.J., Young S. (2008). Low temperature (23 degrees C) increases expression of biofilm-, cold-shock- and RpoS-dependent genes in Escherichia coli K-12. Microbiology (Read).

