# Supplementary material for: Serotype-specific and temperature-dependent biofilm formation in Salmonella: Limited impact of antimicrobial resistance or source
Source: Biofilm. 2026 Mar 17;11:100360. doi: 10.1016/j.bioflm.2026.100360 (PMC13049968; doi:10.1016/j.bioflm.2026.100360)
Supplement: Multimedia component 1 [file mmc1.docx]

**Figure S1**

**
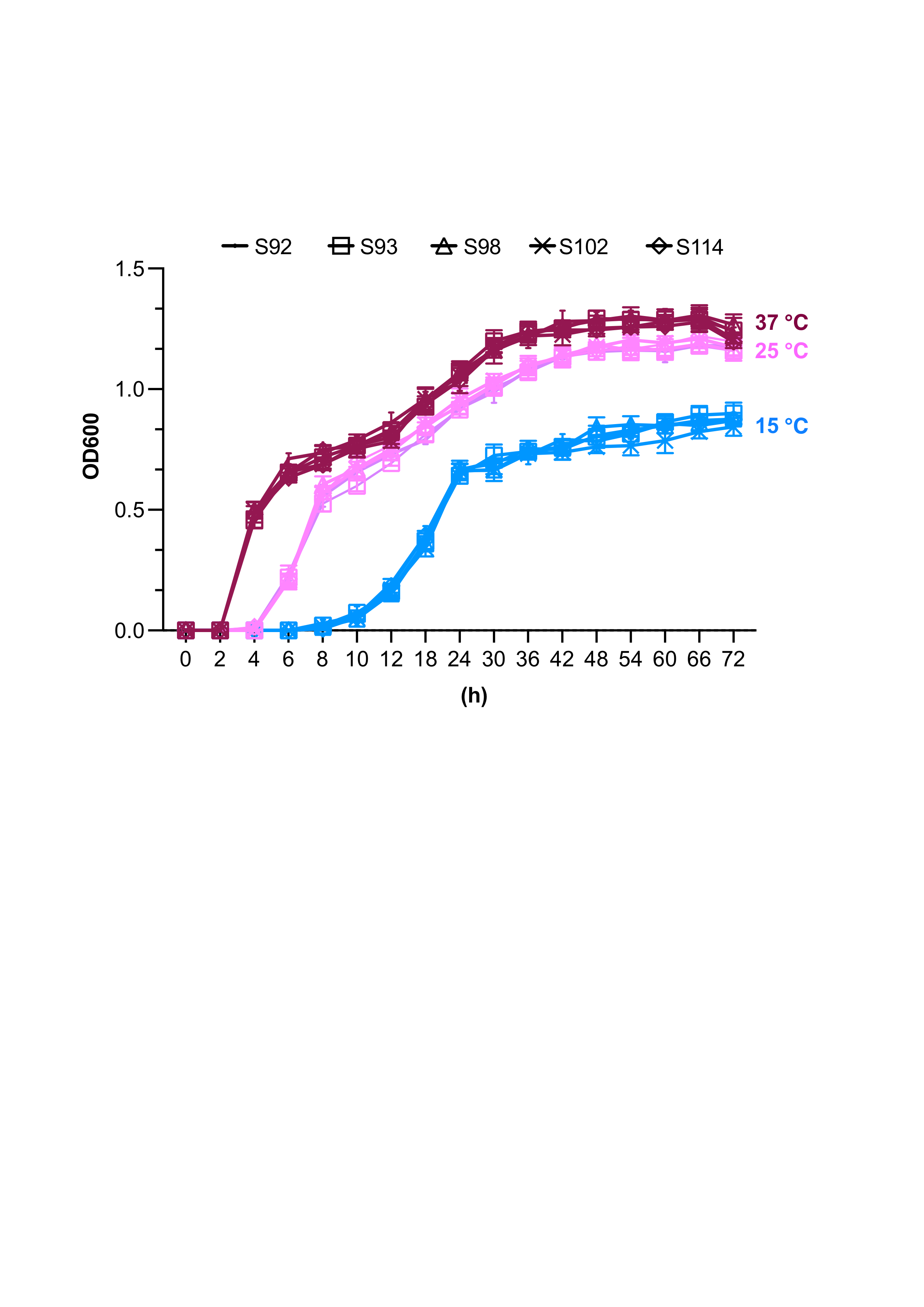
**

**Figure S1 Planktonic growth dynamics of representative *Salmonella* isolates at different temperatures.**

**Figure S2**


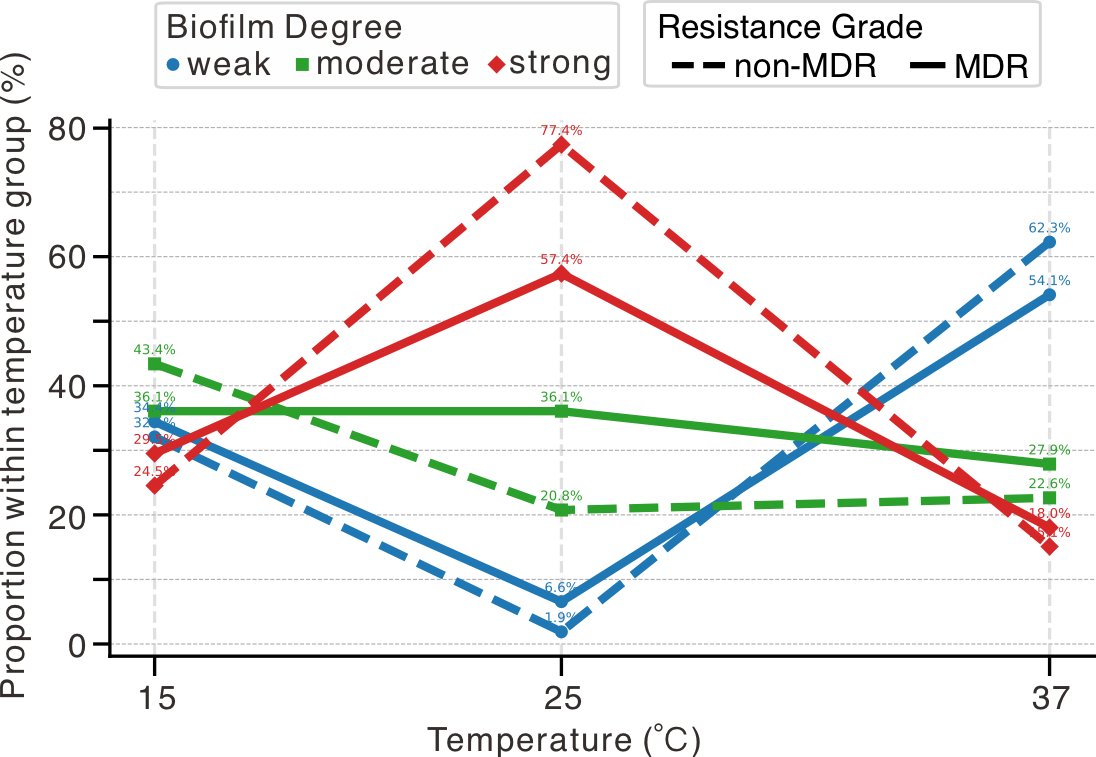


**Figure S2 Line plots illustrating the proportional distribution of *Salmonella* strains by biofilm-forming ability (weak, moderate, strong) across three temperatures (15°C, 25°C, 37°C), separated by antimicrobial resistance grade (MDR vs. non-MDR)**. Colors represent biofilm-forming ability (blue = weak, green = moderate, red = strong), while line styles differentiate resistance grades (solid = MDR, dashed = non-MDR). Notably, strong biofilm formation peaks sharply at 25°C in both resistance groups, while weak biofilm formation dips to a minimum. Percentages are annotated for each group (related to Figure 3E).

**Figure S3**


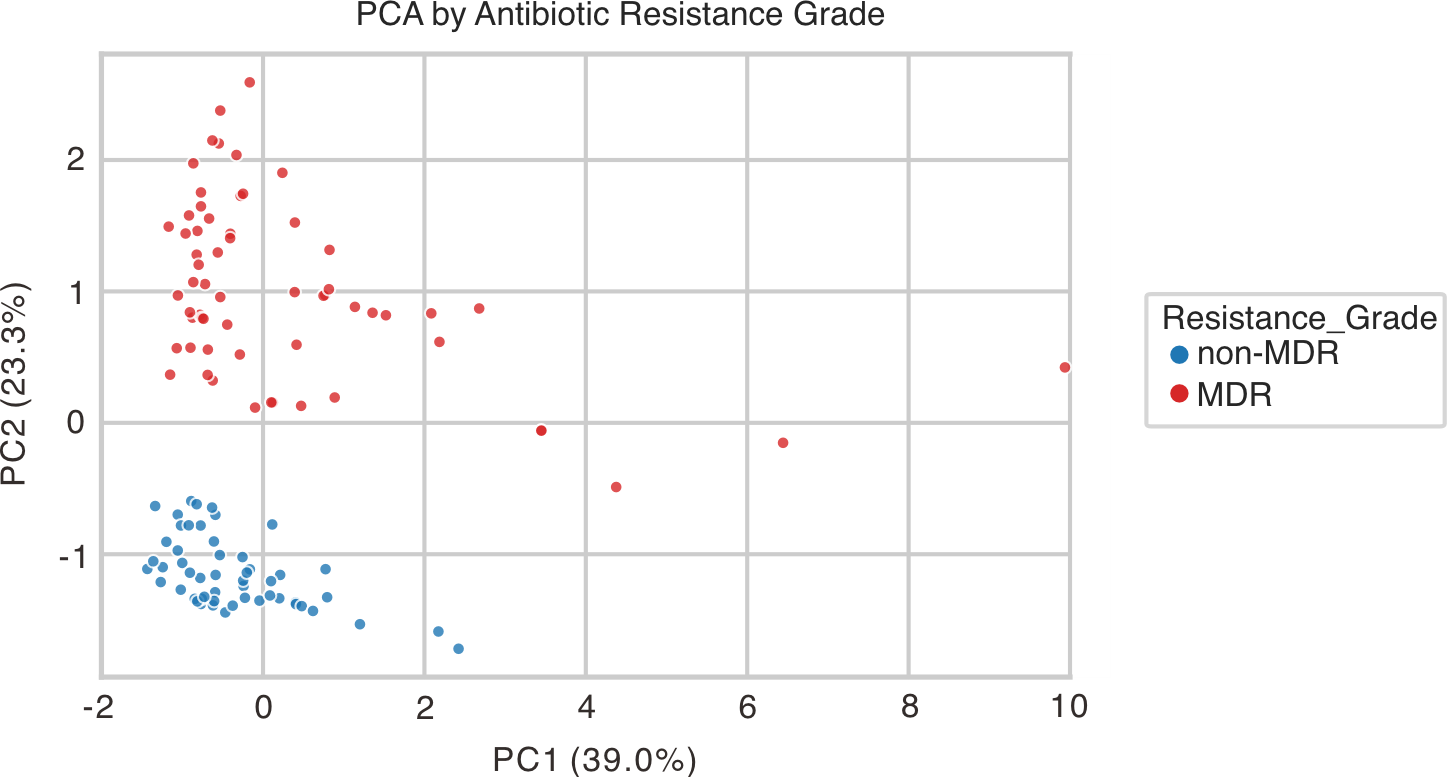


**Figure S3** PCA by Antibiotic Resistance Grade. Principal component analysis (PCA) illustrating distinct clustering patterns of multidrug-resistant (MDR, red) and non-MDR (blue) *Salmonella* strains. PC1 (39.0% variance) separates strains primarily by serovar identity (Albany λ=0.93, Bareilly λ=0.89; Table 1), while PC2 (23.3% variance) reflects antibiotic resistance status (MDR mean PC2 = 0.999 vs non-MDR -1.150; *p* =4.28e-20). The clear spatial segregation in PC space confirms serovar-intrinsic resistance mechanisms independent of biofilm adaptation (related to Figure 3F).

**Figure S4**


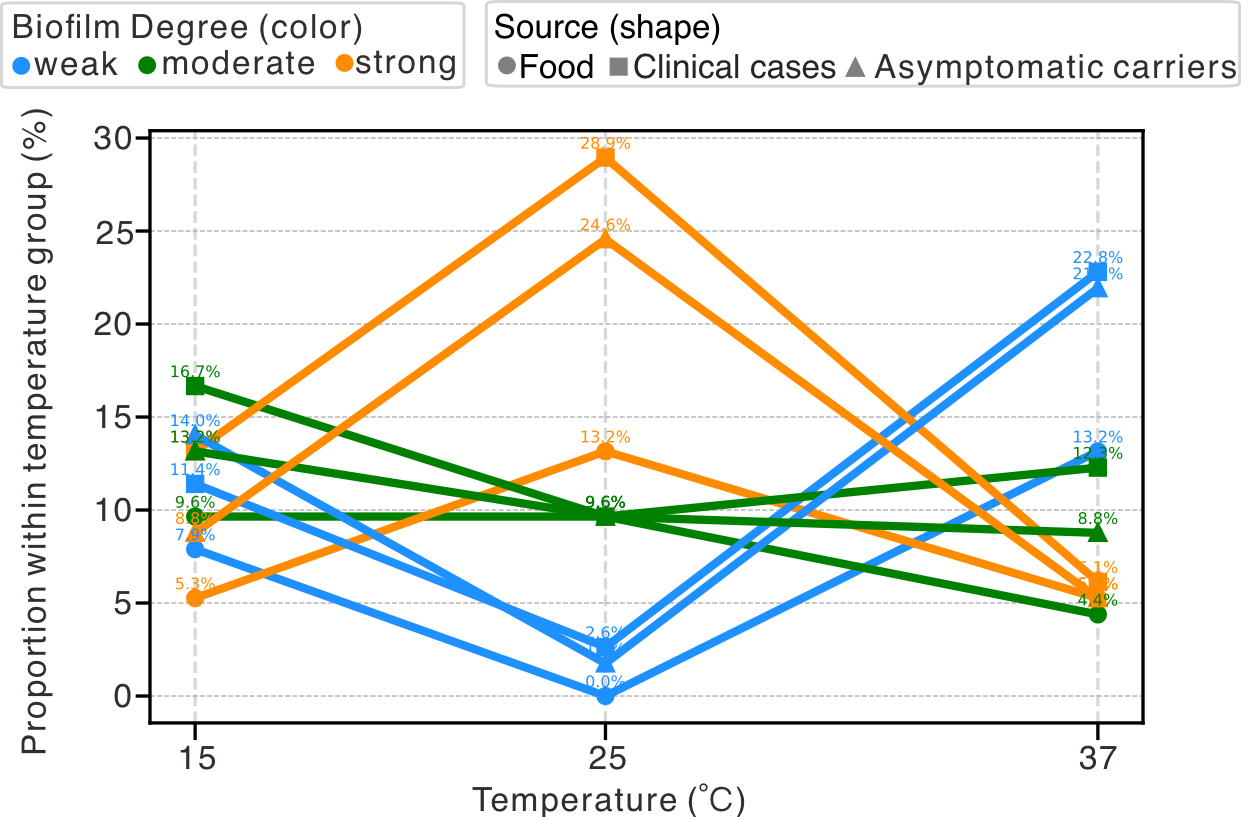


**Figure S4 Line plots illustrating the proportional distribution of *Salmonella* strains by biofilm-forming ability (weak, moderate, strong) across three temperatures (15°C, 25°C, 37°C), separated by different sources (Food, Clinical cases, and Asymptomatic carriers).** Shapes represent sources (circle for Food, square for Clinical cases, triangle for Asymptomatic carriers), and colors indicate biofilm strength (blue for weak, green for moderate, orange for strong) (related to Figure 4C).

**Figure S5**


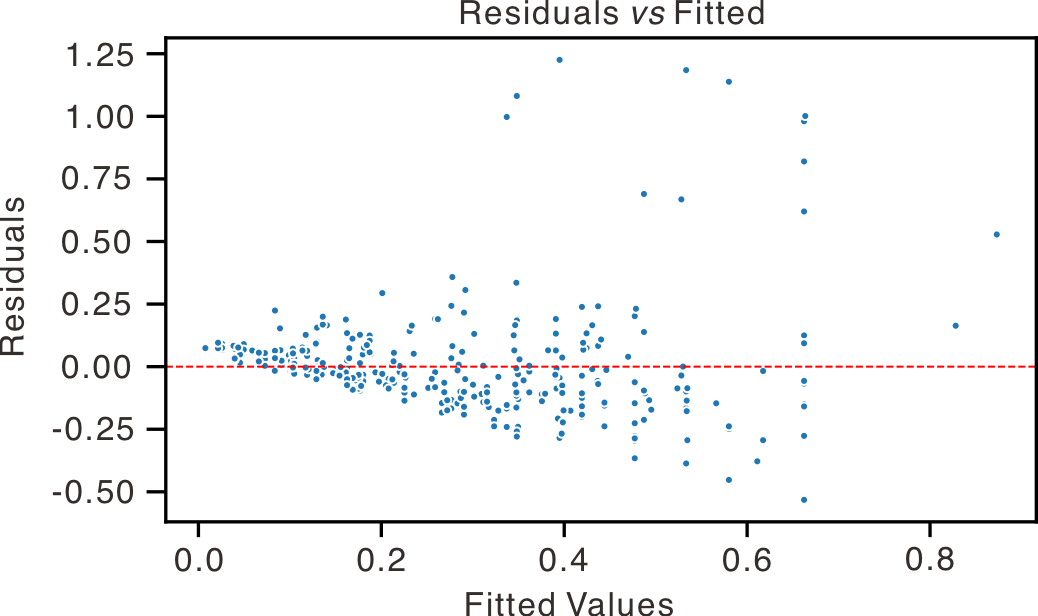


**Figure S5 Scatter plot examines the residuals (observed minus predicted OD_570_ values) versus fitted values (model-predicted OD_570_) to evaluate homoscedasticity and linearity assumptions in regression modeling.** The residual plot reveals subtle heteroscedasticity at extreme fitted values, though variance stability and linearity assumptions remain reasonable across the central prediction range (OD_570_: 0.2-1.5). While no significant nonlinear patterns emerge, model refinement may address dispersion anomalies in outlying regions (related to Figure 5).

**Figure S6**


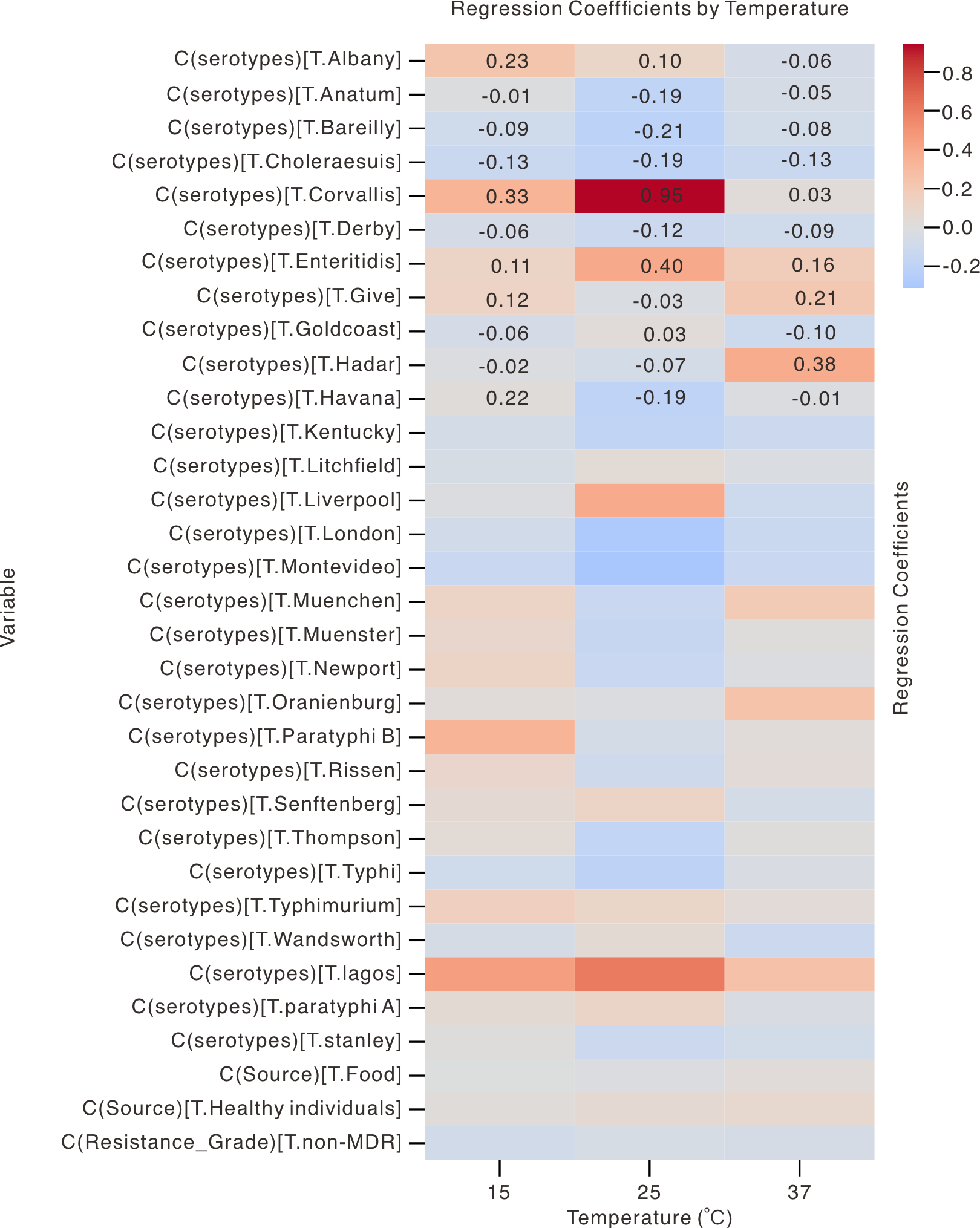


**Figure S6 Heatmap of Regression Coefficients by Temperature** X-axis: Temperature conditions (15°C, 25°C, 37°C); Y-axis: Independent variables in the regression model (including serotypes, source categories, and resistance grades). Color mapping: Blue hues: Negative regression coefficients (β < 0); Red hues: Positive regression coefficients (β > 0) (related to Figure 5).
