# Supplementary material for: Serotype-specific and temperature-dependent biofilm formation in Salmonella: Limited impact of antimicrobial resistance or source
Source: Biofilm. 2026 Mar 17;11:100360. doi: 10.1016/j.bioflm.2026.100360 (PMC13049968; doi:10.1016/j.bioflm.2026.100360)
Supplement: Multimedia component 2 [file mmc2.docx]

**Table S1 List of target genes and specific primer sequences for polymerase chain reaction.**

| Gene | Primers | Sequence（5'-3'） | Products（bp） | Source |
| --- | --- | --- | --- | --- |
| *adrA* | adrA-F | ATGTTCCCAAAAATAATGAATGATG | 1113 | Yin et al. |
|  | adrA-R | TCATGCCGCCACTTCG |  |  |
| *bcsA* | bcsA-F | ATGAGCGCCCTTTCCCG | 2625 | Yin et al. |
|  | bcsA-R | TCATTGTTGAGCCTGAGCCA |  |  |
| *csgB* | csgB-F | TATGATGTTGACAATACTGGGTGC | 419 | This study |
|  | csgB-R | GAATAGCCATATGCGACTGTT |  |  |
| *csgD* | csgD-F | ATGTTTAATGAAGTCCATAGTAGTCATG | 651 | Yin et al. |
|  | csgD-R | TTACCGCCTGAGATTATCGTTT |  |  |
| *csrA* | csrA-F | ATGCTGATTCTGACTCGTCG | 186 | Yin et al. |
|  | csrA-R | TTAGTAACTGGACTGCTGGGA |  |  |
| *csrB* | csrB-F | TCCTCCCACGCTTTTTATTGC | 304 | This study |
|  | csrB-R | TCATATGAAAGTGCGCAAAAAC |  |  |
| *fimH* | fimH-F | ATGAAAATATACTCAGCGCTATTGC | 1008 | Yin et al. |
|  | fimH-R | TTAATCATAATCGACTCGTAGATAGCC |  |  |
| *fliC* | fliC-F | ATGGCACAAGTCATTAATACAAACA | 1488 | Yin et al. |
|  | fliC-R | TTAACGCAGTAAAGAGAGGACG |  |  |
| *glyA* | glyA-F | ATGTTAAAGCGTGAAATGAACAT | 1254 | Yin et al. |
|  | glyA- | TTATGCGTAAACCGGGAA |  |  |
| *igaA* | igaA-F | ATGAGCACCATTCTGATTTTTATAGC | 2133 | Yin et al. |
|  | igaA-R | TCAGATGAGATTTTCCGGAGA |  |  |
| *invA* | invA-F | GTGCTGCTTTCTCTACTTAACAGTG | 2058 | Yin et al. |
|  | invA-R | TTATATTGTTTTTATAACATTCACTGACTTG |  |  |
| *lpfA* | lpfA-F | TTGGTGATACAGACGATGCG | 135 | This study |
|  | lpfA-R | CCAGCGTAGTGGTGGATTTA |  |  |
| *luxS* | luxS-F | ATGCCATTATTAGATAGCTTCGC | 516 | Yin et al. |
|  | luxS-R | CTAAATATGCAATTCCTGCAGTTTT |  |  |
| *misL* | misL-F | AACCCAGTACACCGACATGC | 173 | This study |
|  | misL-R | CAAGACCGTCAGTGCTCCA |  |  |
| *mLrA* | mLrA-F | ATGGCGCTTTACACAATTGG | 732 | This study |
|  | mLrA-R | TTAAACGCCAAGGGGATGAAT |  |  |
| *ompR* | ompR-F | ATGCAAGAGAATTATAAGATTCTGGTG | 720 | Yin et al. |
|  | ompR-R | TCATGCTTTAGAACCGTCCG |  |  |
| *pefA* | pefA-F | ATGAAAAAGAGCATTATTGCTTCC | 519 | Yin et al. |
|  | pefA-R | TTATTTGTAAGCCACTGCGAAA |  |  |
| *pefB* | pfeB-F | TCCATTCACAGCGAGAAGATT | 100 | This study |
|  | pfeB-R | ACCCGTTGTTCACTTCATAGC |  |  |
| *pfs* | pfs-F | ATGAAAATCGGCATCATTGG | 699 | Yin et al. |
|  | pfs-R | TTAGCCATGCGCCAGTTT |  |  |
| *rcK* | rcK-F | ATGAAAAAAATCGTTCTGTCCT | 558 | Yin et al. |
|  | rcK-R | TCAGAACCGGTAACCGA |  |  |
| *rpoS* | rpoS-F | TTGAGTCAGAATACGCTGAAAGTT | 993 | Yin et al. |
|  | rpoS-R | TTACTCGCGGAACAGCG |  |  |
| *sdiA* | sdiA-F | ATGCAGGAAAATGATTTCTTCAC | 723 | Yin et al. |
|  | sdiA-R | TCATATCAGACCTGTCGCC |  |  |
| *sipB* | sipB-F | ATGGTAAATGACGCAAGTAGCA | 644 | This study |
|  | sipB-R | TCTGTGCCTGCTTTAACCGTC |  |  |
| *sipC* | sipC-F | ATGTTAATTAGTAATGTGGGAATAAATCC | 1230 | Yin et al. |
|  | sipC-R | TTAAGCGCGAATATTGCCTG |  |  |
| *sirA* | sirA-F | TTGATCAACGTTCTTCTTGTTGA | 657 | Yin et al. |
|  | sirA-R | TCACTGGCTTGTTAACGTCTC |  |  |
| *srgA* | srgA-F | GTGACGATGAATTATGCCCG | 654 | Yin et al. |
|  | srgA-R | TCAGTCTGCATCAGGGTTACC |  |  |
| *steB* | steB-F | GACGATAAGTTCGCCAAAGAT | 356 | This study |
|  | steB-R | GCATTGCTGATGACGAGGTA |  |  |
| *wcaA* | wcaA-F | ATGACAACAGACAATCCGCT | 843 | Yin et al. |
|  | wcaA-R | TTATCGCCCCCGCAG |  |  |
